# Surface Treatment of Glass Vials for Lyophilization: Implications for Vacuum-Induced Surface Freezing

**DOI:** 10.3390/pharmaceutics13111766

**Published:** 2021-10-22

**Authors:** Francesco Regis, Andrea Arsiccio, Erwan Bourlès, Bernadette Scutellà, Roberto Pisano

**Affiliations:** 1 Department of Applied Science and Technology, Politecnico di Torino, 24 Corso Duca degli Abruzzi, 10129 Torino, Italy; francesco.regis@polito.it (F.R.); andrea.arsiccio@polito.it (A.A.); 2 GSK Vaccines, 89 Rue de l’Institut, 1330 Rixensart, Belgium; erwan.x.bourles@gsk.com (E.B.); bernadette.z.scutella@gsk.com (B.S.)

**Keywords:** freezing, freeze drying, controlled nucleation, surface treatment

## Abstract

Freeze-drying is commonly used to increase the shelf-life of pharmaceuticals and biopharmaceuticals. Freezing represents a crucial phase in the freeze-drying process, as it determines both cycle efficiency and product quality. For this reason, different strategies have been developed to allow for a better control of freezing, among them, the so-called vacuum-induced surface freezing (VISF), which makes it possible to trigger nucleation at the same time in all the vials being processed. We studied the effect of different vial types, characterized by the presence of hydrophilic (sulfate treatment) or hydrophobic (siliconization and TopLyo Si–O–C–H layer) inner coatings, on the application of VISF. We observed that hydrophobic coatings promoted boiling and blow-up phenomena, resulting in unacceptable aesthetic defects in the final product. In contrast, hydrophilic coatings increased the risk of fogging (i.e., the undesired creeping of the product upward along the inner vial surface). We also found that the addition of a surfactant (Tween 80) to the formulation suppressed boiling in hydrophobic-coated vials, but it enhanced the formation of bubbles. This undesired bubbling events induced by the surfactant could, however, be eliminated by a degassing step prior to the application of VISF. Overall, the combination of degasification and surfactant addition seems to be a promising strategy for the successful induction of nucleation by VISF in hydrophobic vials.

## 1. Introduction

Freeze-drying, or lyophilization, is widely used for the long-term storage of pharmaceuticals and biopharmaceuticals. In a freeze-drying cycle, the product is first frozen, before ice is removed by sublimation during primary drying. This drying step is usually carried out at low temperature, thus avoiding harsh conditions for active ingredients. A further drying step, called secondary drying, is performed to allow for the desorption of residual moisture at a higher temperature, typically in the range 10–40 °C.

The product morphology that is formed during freezing influences process efficiency and critical attributes of the final product. The size of the ice crystals formed during freezing corresponds to the porous structure obtained in the dried cake, provided that no shrinkage or collapse occurs. A larger porous structure promotes sublimation, reducing primary drying times [1], but penalizes desorption, therefore making the secondary drying time longer [2]. Moreover, the formation of ice has a dramatic impact on active molecules. Intracellular ice crystals or osmotic changes caused by ice formation may damage tissues or cells, leading to loss of viability [3]. In addition, small ice crystals expose a larger surface area, which may be detrimental for the conformational stability of proteins [4]. Therefore, the formation of large crystals is desired when dealing with protein-based pharmaceuticals, and an accurate control of ice formation is also needed for cell-based products.

Freezing conditions are crucial for both cycle efficiency and product quality, and it would be of paramount importance to have adequate control over the process of ice formation. In this context, the two factors that mostly affect ice crystal size are cooling rate and nucleation temperature [5,6]. A low cooling rate and/or a high nucleation temperature promotes the formation of larger ice crystals, hence smaller specific surface area of the ice/freeze concentrate interface. Cooling rate can easily be adjusted in a typical freeze-dryer. In contrast, the temperature at which the first nuclei start to grow is generally randomly distributed within the batch. To address this problem, several strategies have been developed to trigger ice nucleation at the desired temperature value [7,8,9] and optimize both process control and vial-to-vial homogeneity.

Among such techniques, vacuum-induced surface freezing (VISF) has been shown to improve process efficiency and product homogeneity [10,11,12,13,14,15,16], and in some cases, to have either a negligible [17] or a positive [18] effect on protein recovery. For the application of VISF, the product is first equilibrated at a temperature *T_n_* above the onset of spontaneous nucleation, then the chamber pressure is subsequently reduced and held for a short amount of time *t_n_* (<1 min) to a product-specific value *P_n_* (generally, around 1 mbar [10]). In these conditions, water evaporation from the supercooled solution results in a quick reduction in temperature that induces the formation of ice nuclei at approximately the same time in all vials. After nucleation, pressure is raised back again, and the freezing process continues until complete solidification.

The VISF procedure could also be described as forced nucleation. Indeed, external conditions (temperature and pressure) are created during the application of VISF that will speed up the stochastic nature of nucleation.

The effect of VISF on process performance and product quality has been the subject of detailed investigation, but little is known about the effect of container properties on the application of vacuum-induced nucleation. Among the possible container formats, vials are probably the most commonly used for lyophilized substances [19], and glass is the most widely used material due to its chemical durability, cleanliness, low gas permeability, sterilizability, and transparency [20].

For parenteral drugs, type I borosilicate glass is generally used. Type I glass containers are mainly composed of silicon dioxide (~81%) and boric oxide (~13%), with low levels of non-network-forming oxides such as sodium and aluminum oxides. It is a chemically resistant glass with a low coefficient of thermal expansion [19].

Oxides that cannot enter the structural network of glass are relatively free to migrate from the container to the solution, resulting in leaching. The leaching process is a diffusion-controlled ion-exchange phenomenon involving proton exchange from an aqueous solution for the alkali ions present in glass (e.g., Li^+^, Na^+^, K^+^, Mg^2+^, Ca^2+^, and Mg^3+^). This loss of hydronium ions during leaching leads to a rise in pH value in the product solution, and potential instability of biomolecules [19,21].

During storage of drugs in glass containers, delamination may also occur [22]. Delamination is the result of a chemical attack on the glass surface forming visible or subvisible flakes and particulates that are generally unacceptable [23,24]. Finally, significant quantities of protein or other biomolecules within the solution may adsorb onto the glass of the container, leading to reduced activity and recovery [25,26,27]. To overcome these problems, internal coatings may be applied to the containers [28].

For instance, sulfate-treated vials that underwent a sulfurization treatment with ammonium sulfate salt, are very common. The treatment consists of bringing ammonium sulfate to high temperatures (>490 °C) in such a way that it decomposes. The resulting vapor reacts with surface alkalis (cationic metals), forming water-soluble sodium and potassium salts and displacing calcium with hydrogen. After washing, a silica-enriched layer is formed that acts as a barrier for the further elution of alkali [19,29,30]. Therefore, sulfurization treatment reduces surface alkalinity [31] and suppresses both particle formation phenomena [32] and potential metallic leachates. However, sulfurization may pit the vial surface, enhancing delamination [19,21,33].

Other common surface treatments involve the application of a hydrophobic coating such as siliconization or the deposition of a nonporous Si–O–C–H layer as is the case of the TopLyo vials produced by Schott. Siliconized vials contain lesser quantities of Na and B and more of Si and C than untreated vials, whereas the surface composition of TopLyo vials is dominated by C, Si, and O [34]. Containers displaying an internal hydrophobic coating have some advantages including: (i) a suppression of contact between solution and vial glass [32]; (ii) an effective prevention of alkali elution from glass [31,35], delamination [35,36], and ion leaching; (iii) an improved resistance to nonspecific binding, reducing sample losses, and minimizing protein absorption; and (iv) an almost complete drainage of the product from the vial, allowing for an accurate dosage. The hydrophobic coatings likely prevent the adsorption of hydrophilic excipients/active ingredients, thus being extremely useful in applications where a complete recovery of the material is crucial.

It is evident from this short review that an internal coating can bring important advantages to freeze-dried products. In this work, we studied the effect of such coatings (sulfate-treated, siliconized, and TopLyo vials) on the application of VISF. Different model solutions comprising both an amorphous (sucrose) and a crystalline (mannitol) lyoprotectant as well as a surfactant (polysorbate Tween 80) were considered. We showed that the internal coating affected both the process variables for the application of VISF and final product appearance, displaying a delicate interplay with the formulation.

## 2. Materials and Methods

### 2.1. Materials and Instrumentation

Four vial types (2R size, type I glass) were evaluated: untreated (S−), sulfate-treated (ST), siliconized (S+), and TopLyo (TL). Untreated vials were purchased from Müller + Müller (Holzminden, Germany), sulfate-treated vials were obtained from Nuova Ompi (Piombino Dese, Italy), and TopLyo vials were provided by Schott (Müllheim, Germany). Siliconization was performed by GSK Vaccines (Rixensart, Belgium) on some untreated vials by using a pilot washing and siliconization station (ASVG 100, Groninger, Groninger & Co. GMBH, Crailsheim, Allemagne). A non-ionic emulsion of dimethicone oil-in-water at 35% (silbione emulsion 70001 SP) was applied to the internal surface of vials for this purpose, followed by washing and sterilization (1 h at 260 °C). Helvoet FM 460 igloo stoppers (ultra-pure bromobutyl formulation with extremely high chemical purity and low gas permeability) were used to partially close all vial types during freeze-drying.

Sucrose, mannitol, and Tween 80 were obtained from VWR, Roquette, and NOF Corporation, respectively, and used as supplied. The formulations tested were: water (DW), water + 0.02% *w*/*v* Tween 80 (DW + TW80), 5% *w*/*w* sucrose (Suc), 5% *w*/*w* sucrose + 0.02% *w*/*v* Tween 80 (Suc + TW80), 5% *w*/*w* mannitol (Man), and 5% *w*/*w* mannitol + 0.02% *w*/*v* Tween 80 (Man + TW80). All solutions were prepared in demineralized water and filtered through a 0.22-μm filter before use. The freezing experiments were performed in a pilot-scale freeze-dryer (HETO DW 8030, Thermo Fisher Scientific, Waltham, MA, USA), equipped with a Thermovac TM 101 vacuum meter, and Tempris probes (Tempris GmbH, Holzkirchen, Allemagne) for monitoring the product temperature in selected vials. The Thermovac TM 101 sensor is equipped with a Piezo/Pirani combination sensor, allowing for an accurate pressure measurement in the whole range of 1200–5 × 10^−4^ mbar. This represents an advantage for induced nucleation experiments, where the phenomena involved (bubbling, boiling, nucleation, and blow-up, as detailed later) occur in a wide range of pressure values (from 50 mbar to less than 1 mbar). However, due to the Pirani sensor, measurements below 15 mbar depend on the gas type, and are not accurate when the gas inside the drying chamber is different from nitrogen because of, for instance, water evaporation from the vials. For this reason, the Thermovac TM 101 sensor was used only in freezing experiments, where the number of vials used (20, see following Section 2.2 and Section 2.3) was small enough to result in a negligible rate of evaporation inside the chamber. Moreover, some of the freezing experiments were repeated in a lab scale freeze-dryer (REVO, Millrock Technology, Kingston, NY, USA) equipped with an MKS Baratron pressure sensor, and no noticeable difference was observed from the experiments carried out in the HETO freeze-dryer. Specifically, the values of pressure at which nucleation started to occur in the vials (*P_first_*), and at which all the vials had nucleated (*P_last_*), did not show any remarkable difference when the VISF protocol was applied in the two different freeze dryers. The MKS Baratron sensor used in the REVO freeze dryer cannot measure pressure values above 2 mbar and is therefore not optimal to capture the whole range of phenomena involved in nucleation events. However, its readings are independent of gas type, and allowed us to validate the results obtained with the Thermovac TM 101 sensor. The freeze-drying experiments (Section 2.4) were then performed directly in the REVO freeze-dryer, as the large number of vials used in this case (200), and the correspondingly high evaporation rate, would have risked compromising the Thermovac TM 101 readings.

### 2.2. Determination of the Nucleation Pressure

Twenty vials for each type and formulation (with 0.5 mL filling volume) were loaded onto the freeze-dryer HETO DW 8030, placing them on the central shelf in a staggered way at 4 cm from the edge of the shelf. Tempris sensors were used to monitor the solution temperature inside three vials, and loading was always performed at 4 °C. After 15 min equilibration at 4 °C, shelf temperature was decreased to either −6.5 °C (corresponding to *T_n_* = −5 °C in the solution) in 10 min or to −11.5 °C (corresponding to *T_n_* = −10 °C in the solution) in 15 min. When the vials reached *T_n_* and their temperature was stable, as measured by the Tempris probes, the vacuum pump was switched on to gradually decrease the chamber pressure. The pressure values, *P_first_* and *P_last_*, corresponding to the pressure values at which nucleation was observed in the first or last vial, respectively, were noted down. Atmospheric pressure was then re-established after the last vial nucleated.

Once *P_first_* and *P_last_* had been determined, further experiments (with the same number of vials, filling volume, and cooling ramps previously described) were performed with the objective to determine the time *t_n_* required to induce nucleation at a specific pressure value *P_n_*. For each vial type, formulation, and nucleation temperature (*T_n_*), the value of *P_n_* was calculated as follows:

(1) $$P_{n}=P_{first}-\frac{P_{first}-P_{last}}{c}$$

where *c* is a coefficient initially set to a value of 3. This value of 3 was an empirical choice to discretize the *P_first_* − *P_last_* interval into a reasonable number of points, so that experimental testing could be more straightforward. Once the *P_n_* value was reached, the valve between the condenser and vacuum chamber was closed, and the vacuum chamber remained isolated until nucleation was observed in all vials. The time *t_n_* required to induce nucleation after isolation of the chamber was noted down. After nucleation was triggered in all vials, atmospheric pressure was re-established. If less than 70% of the vials nucleated in a maximum time *t_n_* of five minutes, the experiment was repeated by decreasing the value of *c* by one unit or half a unit. The arbitrary value of 70% was chosen in this work as a compromise between nucleation efficiency (in terms of % of nucleated vials) and mitigation of blow-up phenomena.

### 2.3. Determination of the Influence of the Degasification Process

The effect of degasification was evaluated on the DW and DW + TW80 formulations. Ten TopLyo vials were filled with 0.5 mL of the chosen solution, while another group of ten TopLyo vials was filled with 0.6 mL of the same solution. One Tempris probe was inserted into each group to monitor the temperature of the solution. The group of vials filled with 0.6 mL of solution were placed in a staggered way on the left part of the central shelf of the vacuum chamber at a distance of 4 cm from the front edge of the shelf and were subjected to degasification. In the absence of Tween 80, degasification was performed at 10 °C and 10 mbar for 10 min, followed by a further step at 10 °C and 7 mbar for 20 min. In the presence of Tween 80, degasification consisted of three steps: 10 °C and 10 mbar for 5 min, followed by 10 °C and 7 mbar for 10 min, and eventually 10 °C and 4 mbar for 15 min. The degassing procedure included several stages at different pressures because directly decreasing the pressure to the lower pressure value would cause too intense bubbling, with possible nucleation of the solution inside the vials or leakage of the product from the vials. After completing degasification (which, in all cases, lasted 30 min) atmospheric pressure was re-established, temperature was decreased to 4 °C, and the second group of vials with a filling volume of 0.5 mL was loaded in a staggered way on the right part of the central shelf of the vacuum chamber at a distance of 4 cm from the front edge of the shelf. An additional 0.1 mL of solution in the degassed vials was foreseen to compensate for the evaporation of the solution during degasification.

After loading the second group of vials, the shelf temperature was kept at 4 °C for 15 min, and then lowered to −6.5 °C in 10 min (corresponding to *T_n_* = −5 °C in the solution). When the vials reached *T_n_* and their temperature was stable, the vacuum pump was switched on to gradually decrease the chamber pressure. After the last vial nucleated, atmospheric pressure could be re-established.

### 2.4. Freeze Drying Experiments

The Suc and Suc + TW80 formulations were further subjected to a complete freeze-drying cycle. For this purpose, both untreated and siliconized vials were considered for a total of four different batches (2 formulations × 2 vial types). Each batch comprised 200 vials, with a 0.5 mL filling volume. The freeze-drying experiments were carried out in the REVO freeze-dryer, and two T-type thermocouples were inserted in each batch, inside two selected vials and close to the vial bottom, to monitor temperature evolution. Vials were equilibrated at *T_n_* = −5 °C (corresponding to a shelf temperature of −6.5 °C). The chamber pressure was then decreased to the value of *P_n_* previously determined (see Section 2.2). Once the *P_n_* value was reached, the valve between the condenser and the vacuum chamber was closed, and the chamber isolated for a time *t_n_* (as determined in Section 2.2) before re-establishing atmospheric pressure. Completion of product freezing, and the primary drying and secondary drying phases were then performed as detailed in Table 1.

An additional freeze-drying cycle for the Suc + TW80 formulation inside siliconized vials was also performed, using the same batch setup and cycle details previously described. However, vials were filled with 0.6 mL of solution in this case, and subjected to preliminary degassing, carried out in three steps: 10 °C and 10 mbar for 5 min, followed by 10 °C and 7 mbar for 10 min, and eventually 10 °C and 4 mbar for 15 min. The objective was to investigate the effect of degasification on product appearance after freeze-drying. After the freeze-drying cycles, the vials were visually inspected for the presence of defects due to boiling, bubbling, blow-up, or fogging phenomena. Only vials without any trace of wall staining or cake imperfections were deemed as acceptable.

## 3. Results and Discussion

### 3.1. Effect of Formulation and Vial Type on the Nucleation Pressure

As a first objective of the present work, a series of experiments was carried out to determine the effect of vial type and formulation on nucleation pressure. As detailed in Section 2.2, we first measured the values of *P_first_* and *P_last_* (i.e., the pressures at which the first and last vials nucleated, respectively). The nucleation event was detected by visual inspection, and the nucleation pressure values of the vials containing a Tempris probe were discarded because the probes can act as nucleating agents, altering the nucleation behavior. The results of this first analysis are shown in Table 2.

We observed that the *P_first_* and *P_last_* values were close to 1 mbar, as previously reported [10]. Moreover, neither vial type nor the presence of the surfactant Tween 80 had a marked influence on *P_first_* and *P_last_*. However, *P_first_* and *P_last_* were slightly lower in the presence of sucrose and, even more so, in mannitol. This can be explained considering that the addition of a non-volatile solute to a solution decreases vapor pressure, thus limiting evaporation phenomena. This means that lower chamber pressures are needed in this case to achieve high evaporation rates, and to eventually induce nucleation by VISF.

Another interesting observation is that *P_first_* and *P_last_* were slightly higher at *T_n_* = −10 °C than at *T_n_* = −5 °C (Table 2), albeit the effect was not dramatic (the ANOVA test indicated that the difference was not statistically relevant, with *p*-values above 0.1 for both *P_first_* and *P_last_*). This observation can be explained when thinking about the mechanism of nucleation induction by VISF: a decrease in chamber pressure promotes increased evaporation from the solution, and a consequent decrease in temperature, which eventually promotes ice nucleation. If the starting temperature of the solution (*T_n_*) is lower (−10 °C vs. –5 °C), the temperature decrease needed to induce nucleation is smaller, and the pressure decrease required to induce evaporation is also reduced.

### 3.2. Effect of Formulation and Vial Type on Bubbling and Boiling Phenomena

While performing the first experiments for the determination of nucleation pressure, we noted the occurrence of undesired phenomena (boiling and bubbling) in some vial types and for specific formulations.

Decreasing pressure in the chamber promotes degassing of the solution in the vials. For pressures below 50 mbar, small bubbles start to form between the internal surface of the vials and the solution. These bubbles keep growing at lower pressures, until they burst out at around 10 mbar. This phenomenon, denominated bubbling, stops at pressures in the range of 2–3 mbar.

The other phenomenon induced by pressure reduction is boiling. The boiling point is the temperature at which the vapor pressure of the liquid equals the pressure exerted on the liquid. When chamber pressure decreases below 35–40 mbar, small bubbles begin to form at the bottom of the vials, and the solution may start boiling if too low pressures are reached.

**Figure 1:**
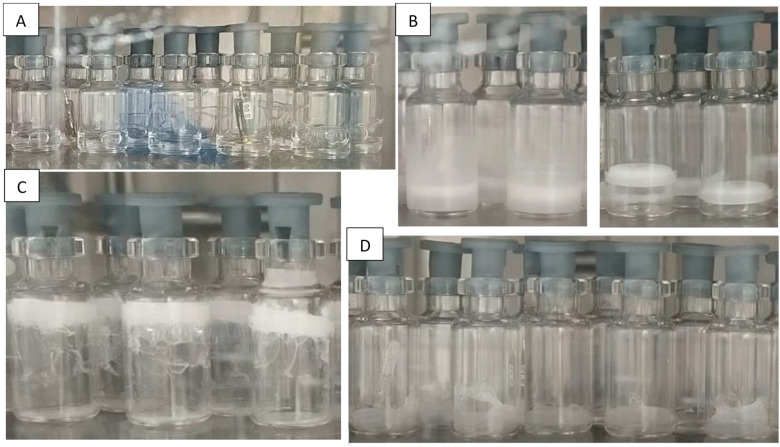
(**A**) Bubbling observed in untreated vials filled with 5% *w*/*w* mannitol + 0.02% *w*/*v* Tween 80 (the chamber pressure was 6 mbar). (**B**) Fogging observed in sulfate-treated vials (**left**), but not in TopLyo vials (**right**) filled with 5% *w*/*w* mannitol, immediately after nucleation. (**C**) Blow up observed in TopLyo vials filled with 5% *w*/*w* sucrose + 0.02% *w*/*v* Tween 80. (**D**) Irregular product in TopLyo vials filled with demineralized water, caused by boiling. All these pictures were taken immediately after nucleation.

Both bubbling and boiling negatively influence the morphology of the cake, often making it unacceptable because of aesthetic defects, and therefore need to be minimized. Defects induced by bubbling and boiling can be observed in Figure 1A,D.

We analyzed both the diffusion and intensity of bubbling/boiling phenomena during our experiments. Regarding diffusion, the following qualitative scale was used: (I) isolated, indicating a phenomenon observed in less than 1/3 of the vials; (W) widespread, for phenomena observed in more than 1/3 of the vials. Regarding intensity, we distinguished between: (W) weak, for a phenomenon of low intensity that only slightly agitates the solution; (M) moderate, for a phenomenon with significant magnitude that moderately agitates the solution; and (S) strong, for a very tumultuous phenomenon that may lead to nucleation and that negatively affects product morphology. The results of this analysis are summarized in Table 3.

It is possible to notice that bubbling was promoted by Tween 80 (Figure 1A, Table 3), and, in a less pronounced way, also by sucrose and mannitol. The reduction in surface tension induced by Tween, and consequent promotion of bubble formation, was probably the basis of the observed fostering of the bubbling phenomena. Sucrose and other sugars were also observed to inhibit bubble coalescence over a concentration range 0.01–0.3 M [38], which may be the basis of our observations. Bubbling was also generally weaker for *T_n_* = −10 °C than for *T_n_* = −5 °C. This may be explained by considering that both oxygen and nitrogen solubility values increase in water at lower temperature, and this reduces their phase separation as bubbles. Finally, it can be observed that bubbling was slightly less widespread and intense in sulfate-treated vials than in untreated ones.

Concerning boiling, this phenomenon seemed to be promoted by the presence of a hydrophobic coating, as was the case in siliconized and, even more so, in TopLyo vials (Figure 1D, Table 3). This occurs because the reduced surface wettability in these conditions, and the consequently weaker interactions between the solution and container, promote a tumultuous release of bubbles at low pressures. We speculate that the reason behind this may be the formation of voids between the solution and the container in conditions of low wettability. These voids act as nucleating spots during boiling, exacerbating this phenomenon. Surface wettability is improved by the surfactant Tween 80, which may prevent the formation of voids between the solution and container, and which was revealed to be extremely effective in suppressing the boiling phenomena (Table 3).

Fogging was also observed in our experiments. Fogging is defined as the spreading of the product and its deposition in spots or as a thin layer on the internal surface of the container above the cake [28,39]. Fogging is promoted by high surface wettability, as such being favored by hydrophilic coatings and surfactants [34,40]. In line with this, we observed fogging in untreated vials and, to an even greater extent, in sulfate-treated vials (Figure 1B) where it is promoted by the low contact angles between the solution and container. The phenomenon was, in contrast, completely absent for the siliconized and TopLyo vials because of their hydrophobic surfaces and reduced wettability [39].

### 3.3. Effect of Formulation and Vial Type on Nucleation Time and Blow Up

As described in Section 2.2, a series of experiments was performed to obtain the combination of pressure (*P_n_*) and holding time (*t_n_*), leading to nucleation in at least 70% of vials. Again, vials containing Tempris probes were not considered for this analysis.

We observed that *t_n_* did not vary much with vial type/formulation and was generally quite short (Table 4). The average value of *t_n_* was on the order of 9 s. Specifically, *t_n_* = 0 means that all vials had already nucleated when the vacuum chamber was isolated from the condenser.

The *P_n_* value was generally comprised between *P_first_* and *P_last_*, according to Equation (1). However, there was one case only where the best combination (*P_n_*, *t_n_*) corresponded to *P_n_* < *P_last_* (Man + TW80 in siliconized vials, *c* = 0.75). In general, we also noted that the *c* factor (Equation (1)) was slightly larger for untreated vials (i.e., *P_n_* closer to *P_first_*) than for other vial types (Table 4), but this effect was not very pronounced. Looking at Table 4, it becomes evident that *P_n_* was not influenced by vial type and was slightly lower only for the mannitol-based formulations.

If pressure release after induction of nucleation occurs too slowly, blow-up may occur (i.e., the frozen product may climb up the surface of the container), driven by a difference in pressure between the vial headspace and bottom (Figure 1C). The holding time *t_n_* determined in our experiments was sufficiently short to avoid, in most cases, the blow-up of the frozen cake. However, some blow-up was observed during the experiments (as evidenced by the presence of the symbol * in Table 4), and we found that this phenomenon was promoted by hydrophobic coatings (siliconized and TopLyo vials). This occurs because the hydrophobic coating results in an extremely homogeneous surface, where friction with the cake is minimized. In the case of blow-up involving solutions with Tween 80, we also noted the formation of bubbles under the lifted cake, leading to unacceptable aesthetic defects (Figure 1C).

### 3.4. Effect of Degasification

Following the procedure described in Section 2.3, the effect of adding a degassing step was also investigated. We focused our attention on the DW and DW + TW80 formulations. Only the TopLyo vial type was considered for this analysis, because we previously found that it was particularly critical in terms of bubbling and boiling phenomena.

**Figure 2:**
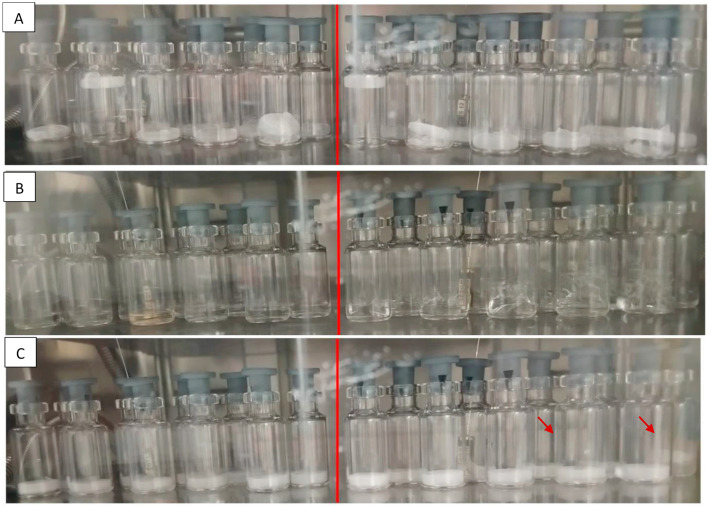
Two groups of TopLyo vials were filled with the DW (**A**) or DW + TW80 (**B**,**C**) formulations. (**A**) Defects due to boiling were observed in both degassed (**left**) and non-degassed (**right**) DW vials. (**B**) In the non-degassed DW + TW80 group (**right**), bubbling phenomena were observed while inducing nucleation. (**C**) The morphology of the DW + TW80 frozen product was acceptable when degassing was performed (**left**), whereas small defects due to bubbling (as indicated by arrows) could be observed in the non-degassed samples (**right**). All these pictures were taken immediately after nucleation.

In the case of the DW formulation, we observed that boiling took place both in the degassed and non-degassed samples when inducing nucleation. The intensity of boiling was lower for degassed vials, but the product aesthetic was still unacceptable (Figure 2A).

The behavior changed upon the addition of the surfactant. During degassing of the solution, we noted moderate bubbling, but the presence of Tween 80 effectively prevented the boiling phenomena. Afterward, when lowering the chamber pressure to induce nucleation, marked bubbling was found to occur only in samples that had not been subjected to degassing (Figure 2B).

We can therefore conclude that the combination of Tween 80 and a preliminary degassing step succeeded in eliminating nucleation defects in hydrophobic containers; the surfactant suppresses boiling due to improved wettability of the internal surface of vials and, in turn, bubbling phenomena are eliminated by degassing.

As can be seen in Figure 2C, the morphology of the frozen cake obtained after nucleation was acceptable for vials subjected to degassing, whereas non-degassed samples presented small defects due to bubbling. This analysis allowed us to conclude that the addition of a degassing step prior to the induction of nucleation is important to improve product appearance and minimize aesthetic defects, especially for vials with an internal hydrophobic coating. We therefore set out to test a whole freeze-drying cycle at the optimized conditions determined from these preliminary experiments.

### 3.5. Optimized Vacuum Induced Surface Freezing Conditions for Freeze-Drying

Following the procedure fully described in Section 2.4, full freeze-drying cycles were performed using the Suc or Suc + TW80 formulations in either untreated or siliconized vials. Siliconized vials were selected because they behave similarly to TopLyo containers, but are more commonly used in industrial processes.

For these freeze-drying cycles, we based the VISF protocol on the results shown in Table 4 (i.e., we employed the values of *P_n_* and *t_n_* previously determined). This is extremely useful for automatizing the controlled-freezing protocol, as the presence of an operator is not required if the values of *P_n_* and *t_n_* are already known. Moreover, this is a necessary requirement for a whole batch, where most of the vials are not in-sight of the operator, and the only way to properly select the nucleation pressure and time is by means of prior knowledge (as in this work), or alternatively, through the use of a suitable monitoring device (for instance, heat flux sensors [41] or infrared thermography [42] that were recently applied to the VISF protocol).

**Figure 3:**
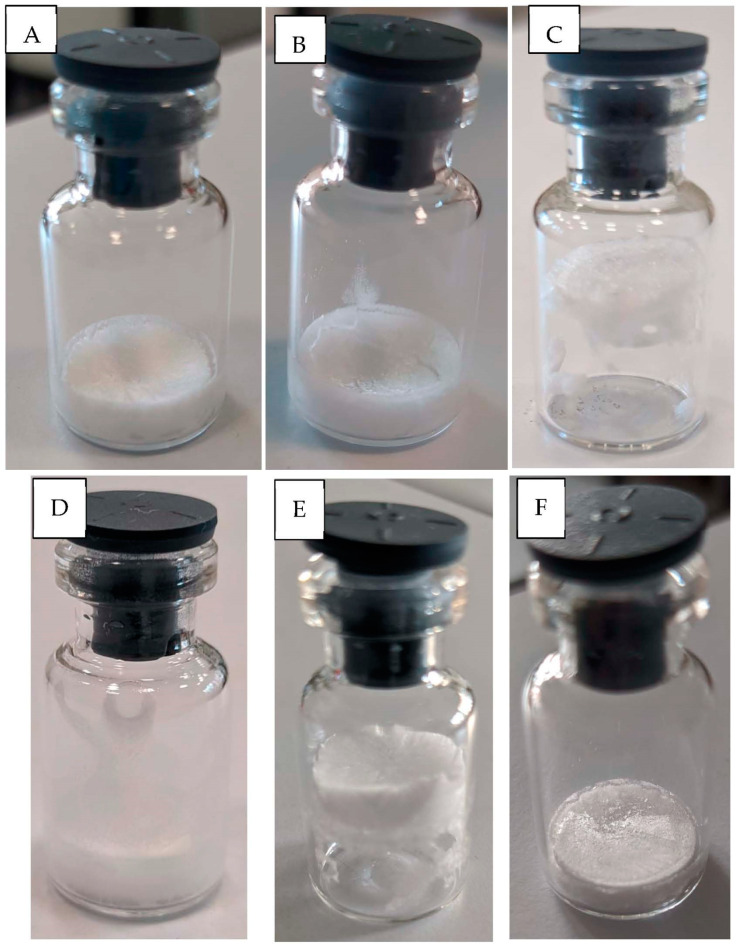
Most freeze-dried cakes of 5% *w*/*w* sucrose in untreated vials were aesthetically acceptable (**A**), but some of them showed fogging (**B**). When 5% *w*/*w* sucrose was dried in siliconized vials, prominent blow-up and boiling were observed (**C**). Freeze-dried cakes of 5% *w*/*w* sucrose + 0.02% *w*/*v* Tween 80 in untreated (**D**) or siliconized (**E**) vials displayed aesthetic defects, mostly due to fogging and bubbling/blow-up, respectively. Freeze-dried cakes obtained for 5% *w*/*w* sucrose + 0.02% *w*/*v* Tween 80 in siliconized vials after degassing (**F**) showed instead a good appearance. All these pictures were taken after secondary drying.

In the case of untreated vials and the Suc formulation, the freeze-dried cakes obtained displayed a fairly good pharmaceutical appearance (Figure 3A). The batch was visually inspected, and about 83.3% of the vials within the batch were found to be aesthetically acceptable (thermocouple-containing vials were excluded from the calculation). Only some minor surface defects due to bubbling or fogging (Figure 3B) were observed.

In the case of siliconized vials and the Suc formulation, only 10.6% of the freeze-dried cakes were aesthetically acceptable, while the remaining 89.4% displayed blow-up or surface defects due to boiling and, marginally, bubbling (Figure 3C). Siliconization results in a hydrophobic surface that resists nonspecific binding and reduces friction with the cake, promoting blow-up.

In the case of untreated vials and the Suc + TW80 formulation, fogging and bubbling phenomena were observed, most likely promoted by the surfactant (Figure 3D). About 36.3% of the freeze-dried cakes were aesthetically acceptable, while the remaining 63.7% displayed surface defects.

The Suc + TW80 formulation inside siliconized vials was freeze-dried both with and without preliminary degassing. In the absence of degassing, blow-up and bubbling were observed, with about 38.7% of the freeze-dried cakes being aesthetically unacceptable (Figure 3E).

When a preliminary degassing step was added, freeze-dried cakes with a good morphology were instead obtained for 99.01% of the vials (Figure 3F) due to the absence of bubbling and boiling phenomena prior to nucleation. The presence of Tween 80 abolished boiling by improving surface wettability, and the degassing process minimized bubbling, as discussed in the previous section.

Overall, the use of vials with a hydrophobic coating (that reduces non-specific binding and minimizes sample loss), in combination with a surfactant (that suppresses boiling phenomena) and a degassing step (that prevents bubbling) seems a promising approach for the application of vacuum-induced surface freezing.

## 4. Conclusions

In this paper, we studied the effect of different surface coatings (sulfate treatment, siliconization, and TopLyo Si–O–C–H layer) on the application of vacuum-induced surface freezing. The study was extended to both amorphous (sucrose) and crystalline (mannitol) formulations, and the presence of a surfactant (Tween 80) was also considered.

We found that the presence of a surface coating did not markedly affect the nucleation pressure (*P_n_*) or time (*t_n_*). However, hydrophobic coatings (i.e., siliconized and TopLyo vials) seemed to promote boiling and blow-up phenomena, while the presence of a hydrophilic inner surface (i.e., untreated and sulphate-treated vials) increased the risk of fogging.

A delicate interplay was found to occur between surface coatings and the composition of the formulation. For instance, the addition of Tween 80 to siliconized and TopLyo vials effectively suppressed the boiling phenomena, but it increased the risk of bubbling. Sucrose and mannitol did not exert any pronounced effect on boiling, but they slightly promoted bubbling events.

We further observed that the addition of a degasification step prior to nucleation could significantly reduce bubbling. We therefore hypothesized that the combination of degassing in the presence of Tween 80, which inhibits boiling, could be a promising strategy for a successful induction of nucleation by VISF. We applied this strategy to the freeze-drying of a sucrose-based formulation in siliconized vials, and eventually obtained elegant cakes, without apparent surface defects. It should, however, be noticed that the drawback of the degasification is the risk of volume loss before freezing, which may be an additional source of heterogeneity inside the batch.

These effects need to be taken into account for a successful cycle development. As a possible future development, it could be interesting to repeat this investigation for other types of container such as ampoules, dual chamber syringes, or cartridges. It could also be instructive to evaluate the application of vacuum-induced surface freezing to super-hydrophilic containers. Fogging phenomena may be exacerbated, but boiling and blow-up phenomena would be eliminated by the high affinity between the aqueous solution and the container surface. Bubbling could be controlled by degassing, and there would be no need to add a surfactant, the presence of which is undesired in some formulations.

## Figures and Tables

**Table 1:** Details of the freeze-drying cycles performed in the present work.

Phase	t, min	T, °C	P, μbar
Loading	-	4	atm
Cooling ramp	10	−6.5	atm
Equilibration	see (1)	−6.5	atm
Nucleation	*t_n_*	−6.5	*P_n_*
Freezing ramp	38	−45	atm
Freezing holding	60	−45	atm
Primary drying ramp	12	−20	100
Primary drying holding	see (2)	−20	100
Secondary drying ramp	120	20	100
Secondary drying holding	400	20	100

(1) Until thermocouples readings were stable at −5 °C. (2) Until completion of sublimation, as determined by comparative pressure measurement [37].

**Table 2:** *P_first_* and *P_last_* values for the different solutions and vial types considered in this work. S−: untreated, ST: sulfate treated, S+: siliconized, TL: TopLyo.

		S−		ST		S+		TL	
Solution	T_n_ °C	P_first_ mbar	P_last_ mbar	P_first_ mbar	P_last_ mbar	P_first_ mbar	P_last_ mbar	P_first_ mbar	P_last_ mbar
DW	−5	1.3	0.7	1.2	0.9	1.2	0.8	1.3	1.2
DW	−10	1.3	0.8	1.3	0.9	1.2	0.9	1.2	0.9
DW + TW80	−5	1.2	0.8	1.2	0.9	1.2	0.9	1.3	0.7
DW + TW80	−10	1.3	0.8	1.3	0.8	1.1	0.8	1.2	0.8
Suc	−5	1.2	0.8	1.1	0.8	1.1	0.8	1.2	0.9
Suc	−10	1.2	0.8	1.1	0.8	1.0	0.8	1.1	0.8
Suc + TW80	−5	1.2	1.0	1.2	0.9	1.2	0.8	1.1	0.7
Suc + TW80	−10	1.2	0.9	1.3	0.9	1.1	0.9	1.2	0.8
Man	−5	1.2	0.7	1.2	0.6	1.1	0.6	1.1	0.8
Man	−10	1.0	0.7	1.3	0.7	1.2	0.8	1.2	0.8
Man + TW80	−5	1.1	0.7	1.2	0.7	1.1	0.8	0.9	0.7
Man + TW80	−10	1.1	0.7	1.2	0.7	1.2	0.8	1.1	0.7

**Table 3:** Diffusion and intensity of bubbling and boiling phenomena for the different solutions and vial types considered in this work. S−: untreated, ST: sulfate treated, S+: siliconized, TL: TopLyo. Diffusion scale: I isolated, W widespread. Intensity scale: W weak, M moderate, S strong.

		S−		ST		S+		TL	
Solution	T_n_ °C	Bubbling	Boiling	Bubbling	Boiling	Bubbling	Boiling	Bubbling	Boiling
DW	−5	-	-	-	-	-	I/W	-	W/S
DW	−10	-	-	-	-	-	I/W	-	W/S
DW + TW80	−5	I/M	-	W/M	-	W/M	-	W/S	-
DW + TW80	−10	I/M	-	W/W	-	W/M	-	W/M	-
Suc	−5	I/M	-	I/W	-	I/M	I/W	I/M	W/S
Suc	−10	I/W	-	I/W	-	I/W	I/W	I/M	W/S
Suc + TW80	−5	I/M	-	I/M	-	W/M	-	W/S	-
Suc + TW80	−10	I/M	-	I/W	-	W/M	-	W/S	-
Man	−5	W/M	-	-	-	I/M	I/W	-	W/S
Man	−10	I/W	-	-	-	I/W	I/W	-	W/S
Man + TW80	−5	W/M	-	I/M	-	W/M	-	W/S	-
Man + TW80	−10	W/M	-	I/W	-	W/M	-	W/S	-

**Table 4:** Nucleation pressure *P_n_*, nucleation time *t_n_*, and *c* factor (see Equation (1)) for the different solutions and vial types considered in the present work. If a * is present in the *t_n_* column, blow up was observed. S−: untreated, ST: sulfate treated, S+: siliconized, TL: TopLyo.

		S−			ST			S+			TL		
Solution	T_n_ °C	P_n_ mbar	t_n_ s	c -	P_n_ mbar	t_n_ s	c -	P_n_ mbar	t_n_ s	c -	P_n_ mbar	t_n_ s	c -
DW	−5	1.1	17	3	1.0	12	2	1.0	1	2	1.2	26 *	1
DW	−10	1.1	15	3	1.1	4	2	1.1	9	3	1.0	3 *	1.5
DW + TW80	−5	1.1	21	3	1.0	5	1.5	0.9	24	1	0.9	28	1.5
DW + TW80	−10	1.0	0	2	1.0	3	1.5	0.9	1	1.5	0.9	1	1.5
Suc	−5	1.0	1	2	0.9	1	1.5	0.9	7 *	1.5	1.0	31 *	1.5
Suc	−10	1.0	2 *	2	0.9	9	1.5	0.9	8 *	2	0.9	0 *	1.5
Suc + TW80	−5	1.0	0 *	1.5	1.0	2	1.5	0.9	1 *	1.5	0.8	7 *	1.5
Suc + TW80	−10	1.0	1 *	1.5	1.0	2	1.5	1.0	1 *	2	0.9	0 *	1.5
Man	−5	0.9	4	2	0.8	2	1.5	1.0	24 *	1.5	0.9	0 *	1.5
Man	−10	0.8	0	2	0.9	58	1.5	1.0	30 *	2	1.0	17 *	2
Man + TW80	−5	0.9	28	2	0.8	4	1.5	0.7	1 *	0.75	0.7	0 *	1
Man + TW80	−10	0.9	21	2	0.8	1	1.5	0.9	2 *	1.5	0.8	3 *	1.5

## Data Availability

Data not included within the manuscript are available upon request.
